# Supplementary material for: Neonatal Exposure to Valproate Induces Long-Term Alterations in Steroid Hormone Levels in the Brain Cortex of Prepubertal Rats
Source: Int J Mol Sci. 2023 Apr 3;24(7):6681. doi: 10.3390/ijms24076681 (PMC10094755; doi:10.3390/ijms24076681)
Supplement: Supplementary file 1 [file ijms-24-06681-s001.zip › ijms-2212382-supplementary.pdf]

International Journal of *Molecular Sciences*

MDPI

Neonatal Exposure to Valproate Induces Long-Term Alterations in Steroid Hormone Levels in the Brain Cortex of Prepubertal Rats

Soon-Ae Kim [1],*, Eun-Hye Jang [1], Jangjae Lee [2,3] and Sung-Hee Cho [2],*

[1] Department of Pharmacology, School of Medicine, Eulji University, Daejeon 34824, Republic of Korea; dmter12@gmail.com

[2] Chemical Analysis Center, Korea Research Institute of Chemical Technology (KRICT), Daejeon 34114, Republic of Korea; jjlee714@krict.re.kr

[3] Department of Chemistry, Korea University, Seoul 02841, Republic of Korea

* Correspondence: sakim@eulji.ac.kr (S.-A.K.); shc0429@krict.re.kr (S.-H.C.); Tel.: +82-42-259-1672 (S.-A.K.); +82-42-860-7713 (S.-H.C.); Fax: +82-42-860-7794 (S.-H.C.)

Supplementary Table S1. Calibration data for steroids in the cerebral cortex

Steroids	Calibration range (ng/g)	Linearity (r^2)	LOD (ng/g)	LOQ (ng/g)
17β-Estradiol	0.03-1	0.995	0.01	0.03
Cortisone	0.1-100	0.997	0.01	0.03
Cortisol	0.1-100	0.996	0.01	0.03
Progesterone	1-100	0.997	0.01	0.03
Allopregnanolone	3-100	0.998	1	3

Supplementary Table S2. Primer information for qPCR

Gene name	Forward sequence (5'→3')	Reverse sequence (5'→3')	Annealing temperature (°C)
Gapdh	AGTGCCAGCCTCGTCTCATA	AGAGAAGGCAGCCCTGGTAA	57
B2m	CGAGACCGATGTATATGCTTGC	GTCCAGATGATTCAGAGCTCCA	58.2
Sdha	TCCTTCCCACTGTGCATTACAA	CGTACAGACCAGGCACAATCTG	58.2
Actb	AAGTCCCTCACCCTCCCAAAAG	AAGCAATGCTGTCACCTTCCC	58.2
Cyp19a1	TCGCAGAGTATCCGGAGGT	TGATACCGCAGGCTCTCGTT	55
Hsd3b1	TGTCATTGATGTCTCACATGTCC	AACTGTGCTGCAGTAGATGAA	59.2
Nr3c1	ACTGCTTCTCTCCTCAGTTCCT	CCTTCCCTTTTGACGATGGC	59.7

Supplementary Table S3. Antibody information for Western blot

Antibody	Dilution	Manufacturer	Cat. No.
β-actin	1:3000	Cell signaling	4970
CYP1B1	1:1000	Invitrogen	PA1-21398
HSD3B1	1:1000	Invitrogen	MA1-46438
GR	1:1000	Cell signaling	12041
HRP-labeled rabbit	1:5000	Invitrogen	31460
HRP-labeled mouse	1:5000	Invitrogen	31430

Supplementary Table S4. Concentrations of steroids in neonatal VPA exposed 4 weeks old rat cortex and their controls

**Sex**	**Steroids**	**Concentration[a] (mean ± SD)**		***P*-value**
		**Controls (n = 12)**	**VPA (n = 11)**	**Controls : VPA**
Female	Cortisol	6.1 ± 1.7	9.0 ± 2.8	< 0.03
	Cortisone	2.3 ± 0.8	3.9 ± 1.3	< 0.01
	Progesterone	10.0 ± 3.2	6.3 ± 1.8	< 0.007
	Allopregnanolone	11.6 ± 2.3	8.1 ± 2.1	< 0.005
Male	Cortisol	5.9 ± 1.8	10.2 ± 3.9	< 0.002
	17β-estradiol	0.20 ± 0.08	0.12 ± 0.03	< 0.004

[a]Concentration was expressed as ng/g (mean ± SD).

Supplementary Table S5. Concentrations of steroids in the cerebral cortex of prepubertal VPA exposed 8 weeks old rat and their controls

Sex	Steroids	Concentration[a] (mean ± SD)		*P*-value
		Controls (n = 10)	VPA (n = 10)	Controls : VPA
Female	Cortisol	6.4 ± 2.4	7.6 ± 2.8	NS[b]
	Cortisone	2.4 ± 0.7	2.9 ± 1.5	NS
	Progesterone	12.1 ± 3.4	7.5 ± 2.7	< 0.01
	Allopregnanolone	12.4 ± 3.1	7.6 ± 1.4	< 0.001
Male	Cortisol	7.1 ± 2.5	8.7 ± 4.2	NS
	17β-estradiol	0.26 ± 0.10	0.14 ± 0.07	< 0.01

[a]Concentration was expressed as ng/g (mean ± SD).

[b]NS, not significant

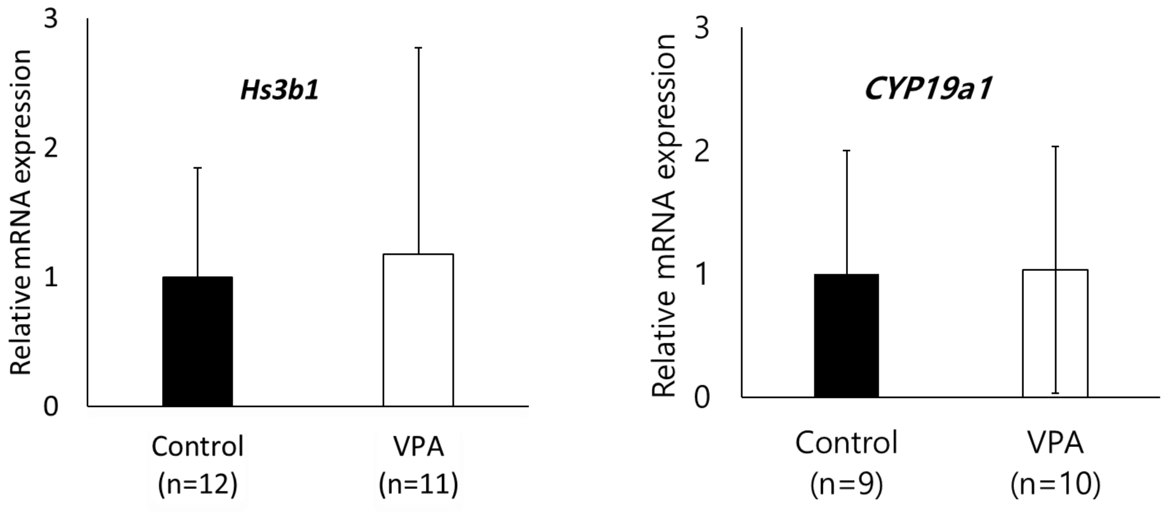

Supplementary Figure S1. Relative mRNA expression of *CYP19a1* at female and *Hsd3b1* at male in prenatal VPA-exposed 4 weeks old rat cortex.

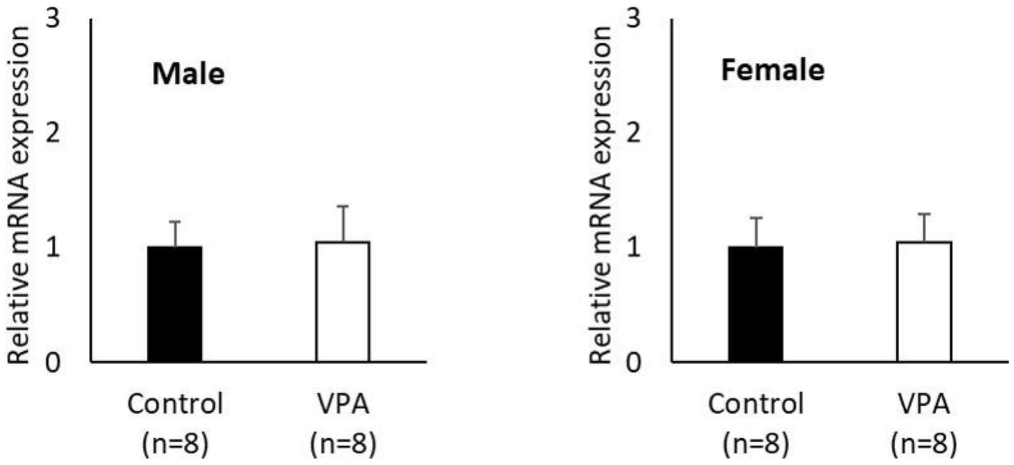

Supplementary Figure S2. Relative mRNA expression of *Nr3c1* in prepubertal VPA-exposed 8 weeks old male and female rat cortex.
